# Understanding sprint phase-specific training stimuli: a cluster analysis approach to overload conditions

**DOI:** 10.3389/fspor.2024.1510379

**Published:** 2024-12-10

**Authors:** Pedro Jiménez-Reyes, Roland van den Tillaar, Adrián Castaño-Zambudio, Sam Gleadhill, Ryu Nagahara

**Affiliations:** ^1^ Sport Sciences Research Centre, Rey Juan Carlos University, Madrid, Spain; ^2^ Department of Sports Sciences and Physical Education, Nord University, Levanger, Norway; ^3^ University of South Australia, Adelaide, Australia; ^4^ National Institute of Fitness and Sports in Kanoya, Kanoya, Japan

**Keywords:** resistance-based sprint training, overload conditions, ground reaction forces, dynaspeed, running speed, sprint phases, force plate

## Abstract

**Introduction:**

This study analyzed the impact of various overload conditions on sprint performance compared to free sprinting, aiming to identify the loading scenarios that most closely replicate the mechanics of unresisted sprints across the full acceleration spectrum. While velocity-based training methods have gained popularity, their applicability is limited to the plateau phase of sprinting.

**Methods:**

To address this limitation, we employed cluster analysis to identify scenarios that best replicate the mechanical characteristics of free sprinting across various overload conditions. Sixteen experienced male sprinters performed sprints under six conditions: unresisted, overspeed (OS) and four overloaded conditions inducing a velocity loss (VL) of 10%, 25%, 50% and 65% using a resistance training device with intelligent drag technology. Ground reaction forces and spatiotemporal parameters were recorded for all steps using a 52-meter force plate system for all sprint conditions.

**Results:**

Cluster analysis revealed four distinct groups aligning with established sprint phases: initial contact, early-acceleration, mid-acceleration, and late-acceleration. Results showed that heavier loads prolonged the mechanical conditions typical of early-acceleration and mid-acceleration phases, potentially enhancing training stimuli for these crucial sprint components of sprint performance. Specifically, VL50 and VL65 loads extended the early-acceleration phase mechanics to steps 7–8, compared to steps 2–4 for lighter loads. Conversely, lighter loads more effectively replicated late-acceleration mechanics, but only after covering substantial distances, typically from the 11- to 29-meter mark onwards.

**Discussion:**

These findings suggest that tailoring overload conditions to specific sprint phases can optimize sprint-specific training and provide coaches with precise strategies for load prescription. These insights offer a more nuanced approach to resistance-based sprint training by accounting for every step across all acceleration phases, rather than focusing solely on the plateau phase, which accounts for only 20–30% of the steps collected during initial contact to peak velocity depending on the analyzed overload condition.

## 1 Introduction

Sprint performance, a crucial component in many sports, heavily depends on the ability to generate propulsive force across a range of speeds. Assessment and modelling of this mechanical capacity often rely on the force-velocity (F-v) relationship, especially in scenarios involving sprint acceleration and other maximal effort movements. Widely validated and frequently used in practice (1–6), the Fv profile provides valuable insights into an individual's ability to generate force under different speed conditions throughout a sprint.

In sprinting, forward acceleration is predominantly influenced by the ground reaction forces (GRFs) generated by the athlete. The interaction between the vertical and horizontal components of these GRFs creates a resultant vector that varies throughout different phases of the sprint. A critical factor in this process is the athlete's “technical ability,” often quantified by the ratio of forces (RF). The RF represents the proportion of the step-averaged resultant ground reaction force vector that is directed horizontally (FH) relative to the total applied force (7). The orientation and magnitudes of the force vector, as indicated by the RF, serves as a key mechanical parameter, influencing both the efficiency of acceleration (7, 8) and performance when approaching maximum speed (7, 9). Consequently, a comprehensive understanding of force production and its directional components across varying speed conditions during sprint acceleration is essential for optimizing performance and assessing the kinetic effects of different training protocols.

A significant challenge in training to improve ratio of forces has been determining how to effectively stimulate different phases of a sprint, particularly under varying force-velocity conditions. Among the most prevalent methods for enhancing sprint performance is sprint-specific resisted training, which involves running against varying resistance loads to target specific F-v characteristics (10). Resisted runs are well-known for creating the desired force application context, which is necessary to stimulate specific scenarios during a sprint. In the existing literature, two major context categories have been identified as relevant to the application of anteroposterior force in the context of a standing maximal sprint. These categories are high force-low velocity condition, and low force-high velocity condition, which are observed to occur successively throughout the run.

Historically, the speed of displacement has been a cornerstone for identifying distinct phases of sprinting. More recently, it has also been used as a criterion for prescribing training loads due to its integration of various underlying explanatory variables (8, 11, 12). The velocity-loss approach has gained popularity as an alternative to traditional methods based solely on relative body weight percentages which may not fully account contextual or individual factors -such as variations in friction coefficients or force production capabilities- that can influence the orientation of sprint training stimuli (13–15). The introduction of motorized towing systems (e.g., Musclelab Dynaspeed, 1080 Sprint) has provided coaches with greater control and precision, enabling them to create specific conditions that stimulate athletes and induce sprint-specific overloads, expanding the possibilities beyond traditional loaded sleds, parachutes and elastic bands (3, 16, 17).

It is now recognized that training with specific loads can significantly impact not only the kinetic (force orientation and application) but also kinematic parameters underlying sprint performance. Recent studies have increasingly focused on the effects of sprint resistance training, exploring how different loads affect underlying components such as spatiotemporal kinematics (17, 18), segmental disposition (19, 20), or electromyographic activity (11). However, debate persists over the use of light vs. heavy loads, due in part to the differences between unresisted and resisted sprint conditions, which are not always fully explained (21–23). Much of this research is also based on cross-sectional studies, which often focus on isolated steps within specific sprint phases rather than analysing full acceleration, potentially limiting the understanding of a such a dynamic mechanical context. Additionally, findings from acute overload effects are sometimes interpreted as potential long-term adaptations, though these projections remain unconfirmed under longitudinal studies (20). Although load specificity is a well-established concept in the broader field of resistance training (24–26), its application in sprint training remains underexplored, with limited experimental research offering a step-by-step analysis of kinetic and kinematic changes across different sprint conditions.

Despite these ongoing debates, a primary goal of sprint resistance training is to achieve specificity by manipulating training loads to replicate the kinetic and kinematic conditions of unresisted sprints at various velocities (22, 23, 27, 28). It is well-documented that sprint mechanics remain largely unaffected between free and overloaded conditions when running velocities are similar (29). However, from a practical perspective, this approach only permits accurate characterization of training stimuli during phases where velocity remains constant, such as the plateau phase. Therefore, this study aims to analyze the impact of various loading conditions on sprint performance in comparison to free sprinting. Specifically, the research seeks to identify the loading scenarios that most closely replicate the mechanics of unresisted sprints across the full acceleration spectrum, thereby providing practical guidelines for coaches in selecting appropriate loads for sprint-specific overload training.

## 2 Material and methods

Sixteen experienced male sprinters competing at state level volunteered to participate (age: 20.5 ± 1.4 years; stature: 1.75 ± 0.06 m; body mass: 68.3 ± 6.4 kg; 100 m personal best, 11.31 ± 0.38 s). Participants were informed about the testing procedures and potential risks, and written consent was obtained prior to the study. They were advised to avoid any fatiguing lower-body activities, including resistance training, sprinting, or high-intensity interval running, for 72 h before testing to ensure they arrived in a non-fatigued state. The study adhered to current ethical guidelines, was approved by the institutional research ethics committee (#11–54), and followed the latest revision of the Declaration of Helsinki.

### 2.1 Procedure

Two familiarization sessions and one testing session were conducted, spaced by a minimum of two days. Participants, who had extensive experience with resisted and assisted sprint training and had engaged in similar studies, underwent these sessions. The dynaSpeed robotic pulley system (Ergotest Technology AS, Stathelle, Norway), which utilizes a cable mechanism attached to a waist-secured belt, provided resistance and assistance. During the familiarization, the protocol included 50 m sprints with at least five minutes of passive recovery, and 30 m sprints with three minutes of passive recovery. Each session also involved five 20 m resisted sprints with variable pulling resistance and two 50 m assisted sprints with 5 kg of pulling assistance, facilitating adaptation to the robotic pulley system prior to the main testing session.

The main testing session, dedicated to data collection, was conducted on a 110 m long indoor athletic track outfitted with a force platform system for 52 m. This system, consisting of 54 units (models TF-90100, TF-3055, TF-32120, Tec Gihan, Uji, Japan), operated at a sampling frequency of 1,000 Hz. The protocol involved initial sprints of 50 m unresisted, followed by two 30-m sprints with active resistance at 10%, 25%, 50%, and 65% velocity loss, provided by dynaSpeed (Ergotest Technology AS). These velocity losses were calculated based on a linear regression analysis of sprints with different resisted loads after the first familiarization session and tested in the second session. Overspeed condition (OS) was evaluated using the same methodology, ensuring a 5% increase in top-speed. These sprints were either completed over the designated distance or continued until the plateau phase stabilized, defined in this study as the portion of the sprint where velocity ranged between 90%–100% of each participant's top speed. The order of resistance levels administered randomly. Warm-ups were self-selected and tailored by the participants (ranging from 35 to 50 min), who leveraged their experience to optimize performance and minimize injury risk. This standardized routine included 5 min of low-intensity jogging, followed by upper and lower limb dynamic stretches, progressing to sprint drills (e.g., high knee runs, skips, bounds), and concluding with three progressive sprints building to 95% of maximum effort. A minimum of five minutes of passive recovery was mandated between sprints. Sprint trials commenced from a three-point start. Participants wore their personal spiked racing shoes and maintained consistent attire across all trials.

### 2.2 Data analysis

Captured kinetic data at a sampling rate of 1,000 Hz. Data integrity was ensured by processing the raw outputs through a digital 50 Hz low-pass, fourth-order Butterworth filter. Spatiotemporal variables and GRFs at every step were calculated in reference to previous studies (30, 31), using the following procedures. Step-to-step contact and flight times were determined using a 20 N threshold for vertical GRFs to identify when the foot made or left ground contact. Step duration was the time between two consecutive foot strikes, and step frequency was calculated as its inverse. Foot placement corresponded to the center of pressure (CoP) at the midpoint of the contact phase. Step length was measured as the anteroposterior distance between CoP positions of consecutive steps. Running speed was determined by multiplying step length by step frequency. Additionally, mean anteroposterior and vertical forces during the contact phase were assessed for each step. RF was calculated by dividing the FH by the total force applied during each step. Finally, effective vertical mean force and impulse were calculated by subtracting body mass from the vertical force and integrating over the contact phase duration (32, 33). All GRFs and impulse variables were divided by body mass. Relative values were expressed as a percentage of the maximum value observed during the free sprint condition, calculated using the formula: (value under load/maximum value without load) × 100. For instance, if the step length under load is 1.8 m and the maximum step length during unresisted sprint is 2 m, the resulting relative value would be 90%.

### 2.3 Statistical analysis

#### 2.3.1 Cluster analysis

K-means clustering was applied to every step to classify the dataset into distinct groups based on five key biomechanical variables: ratio of force, relative mean horizontal force, relative mean vertical force, relative contact time, and relative step length. Prior to clustering, variables were standardized using z-scores to ensure equal contribution and prevent any single variable from disproportionately influencing cluster formation. The optimal number of clusters was determined using the elbow method, which involved identifying the point where the reduction in within-cluster sum of squares began to plateau. Silhouette analysis was then used to confirm the cohesiveness and separation of the clusters. To further validate cluster stability and reliability, we performed cross-validation by randomly selecting two subsets of approximately 800 steps each (representing around 30% of the entire dataset). Each subset was analyzed independently, using the same clustering procedures. These additional analyses yielded the same optimal number of clusters, supporting the robustness of the clustering approach applied to the full dataset.

#### 2.3.2 Inter-cluster statistical comparisons

Once non-normality of the data was confirmed through Levene's test for each analyzed variable (*p* < .001), a series of Kruskal-Wallis tests were conducted to assess differences in selected biomechanical parameters across four clusters. Each test was followed by Dunn's post-hoc pairwise comparisons to identify specific inter-cluster differences, with significance set at *p* < 0.05. Descriptive statistics were calculated as group-averaged minimum and maximum values of key biomechanical variables across different loading conditions within each cluster.

## 3 Results

### 3.1 Cluster identification

A four-cluster solution was chosen based on a balance between fit statistics and interpretability. The overall fit statistics for the clustering solution were as follows: the coefficient of determination (R^2^) was 0.888, the Akaike Information Criterion (AIC) was 962.190, and the Bayesian Information Criterion (BIC) was 1070.360. The average silhouette score, which measures the cohesion and separation of clusters, was 0.540, indicating reasonably well-structured data.

### 3.2 Cluster characteristics

**Figure 1:**
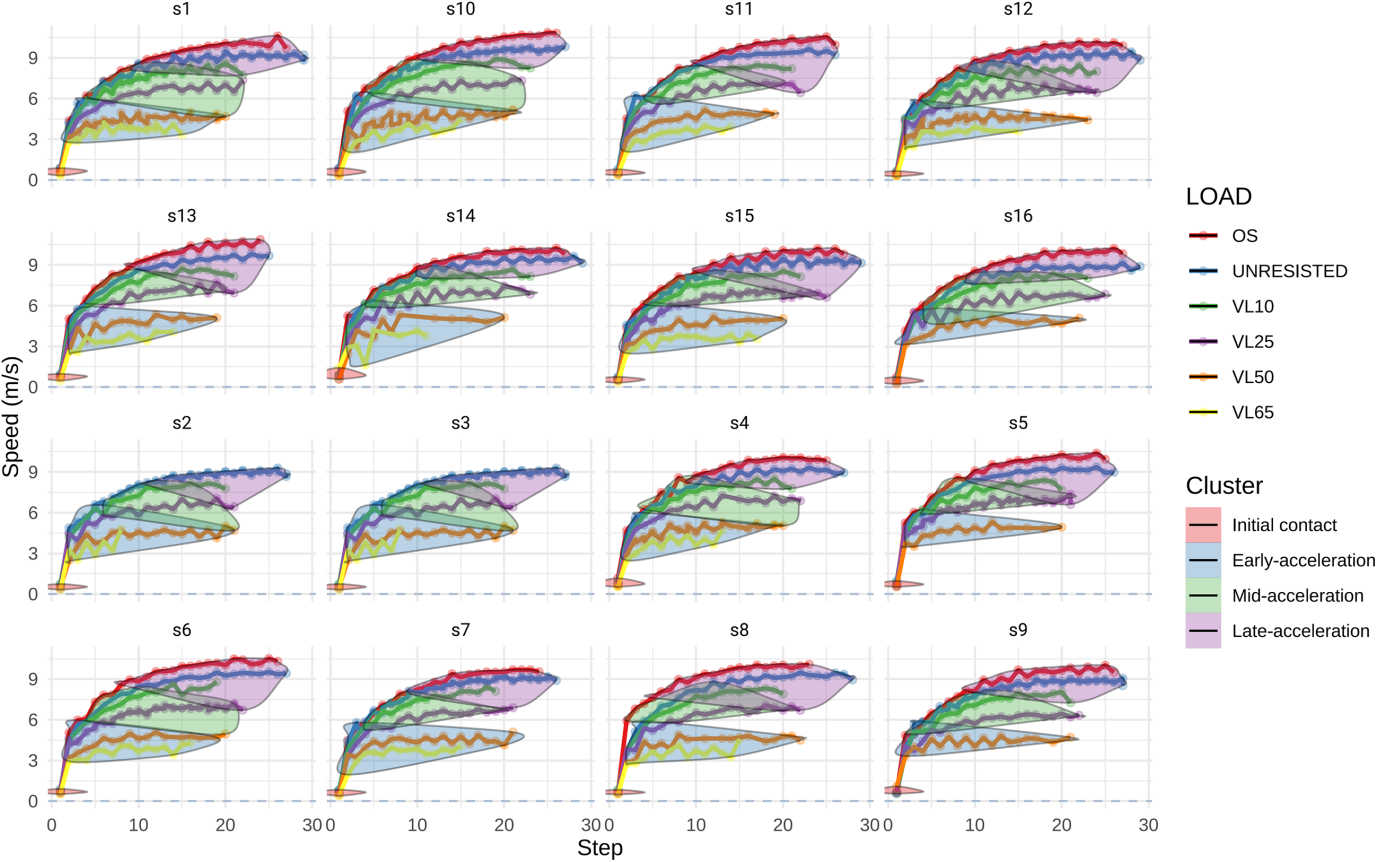
Cluster representation of overloading conditions in relation to individual acceleration performance.

The cluster sizes ranged from 109 to 771 steps, with Cluster 4 having the largest group (771) and Cluster 1 the smallest (109). The proportion of within-cluster heterogeneity explained varied across clusters, with Cluster 2 explaining the most heterogeneity (35.3%) and Cluster 4 the least (15.9%). The within-cluster sum of squares, a measure of cluster compactness, showed that Cluster 1 had the highest value (325.423), while Cluster 4 had the lowest (146.556). The silhouette scores for individual clusters ranged from 0.467 (Cluster 2) to 0.635 (Cluster 4), suggesting that Cluster 4 had the best separation from other clusters (see Table 1). Visual representation for identified clusters can be observed in Figure 1.

**Table 1:** Cluster information.

Cluster	Associated phase	Size	Explained proportion within-cluster heterogeneity	Within sum of squares	Silhouette score
1	Initial contact	109	0.159	146.556	0.635
2	Early-acceleration	763	0.353	325.423	0.551
3	Mid-acceleration	716	0.271	249.558	0.467
4	Late-acceleration	771	0.218	200.652	0.596

### 3.3 Differences across clusters

The Kruskal-Wallis test revealed significant differences between the clusters for each performance-related parameter analyzed. Post-hoc analysis further indicated that the clusters differed significantly from one another, with the exception of relative horizontal force, where no significant difference was observed between clusters 4 and 1 (*p* > 0.05). A detailed breakdown of these findings is provided in Table 2.

**Table 2:** Differences across clusters for biomechanical parameters.

	Kruskall-Wallis	Cluster differences
Ratio of forces (%)	*χ*^2^ (3, *N* = 1,650) = 1,466.748, *p* < .001	1 > 2 > 3 > 4
Relative horizontal force (%)	χ^2^ (3, *N* = 1,650) = 1,413.08, *p* < .001	1; 2 > 3 > 4
Relative vertical force (%)	χ^2^ (3, *N* = 1,650) = 1,440.686, *p* < .001	1 > 2 > 3 > 4
Relative CONTACT TIme (%)	χ^2^ (3, *N* = 1,650) = 1,294.251, *p* < .001	1 > 2 > 3 > 4
Relative step length (%)	χ^2^ (3, *N* = 1,650) = 1,467.299, *p* < .001	1 > 2 > 3 > 4

### 3.4 Step characteristics per cluster

**Figure 2:**
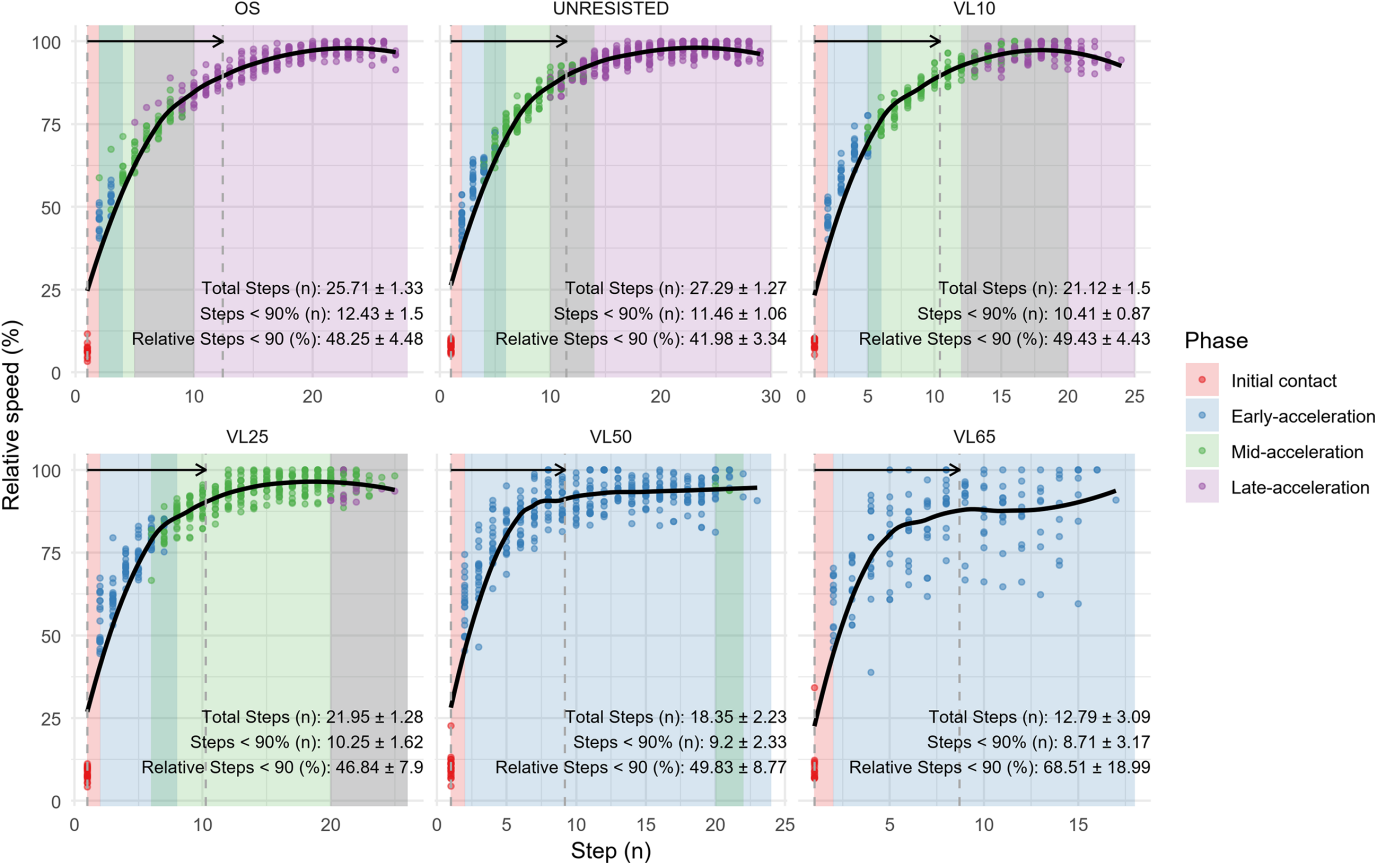
Steps to the plateau phase across different loading conditions.

Table 3 presents the minimum and maximum values of key biomechanical variables across different loading conditions within distinct clusters. Visual representation of these ranges is shown in Figure 2.

**Table 3:** Mean ranges for biomechanical variables across clusters.

			Distance	Step	Speed	Contact time	Step length	Horizontal force	Vertical force	Ratio of force
CLUSTER 1. Initial Contact	OS	Absolute	0.0–0.0	1.0–1.0	0.7–0.7	0.9–0.9	0.7–0.7	3.9–3.9	11.8–11.8	–
		Relative	0.0–0.0	3.9–3.9	7.2–7.2	136.9–136.9	29.6–29.6	56.4–56.4	44.0–44.0	31.1–31.1
	UNRESISTED	Absolute	0.0–0.0	1.0–1.0	0.8–0.8	0.64–0.64	0.5–0.5	5.9–5.9	12.1–12.1	–
		Relative	0.0–0.0	3.7–3.7	7.9–7.9	96.8–96.8	23.6–23.6	85.5–85.5	45.0–45.0	43.9–43.9
	VL10	Absolute	0.0–0.0	1.0–1.0	0.7–0.7	0.64–0.64	0.5–0.5	6.3–6.3	12.1–12.1	–
		Relative	0.0–0.0	4.8–4.8	7.8–7.8	99.1–99.1	23.2–23.2	91.2–91.2	45.3–45.3	46.2–46.2
	VL25	Absolute	0.0–0.0	1.0–1.0	0.6–0.6	0.69–0.69	0.4–0.4	6.8–6.8	12.2–12.2	–
		Relative	0.0–0.0	4.5–4.5	6.2–6.2	105.0–105.0	19.8–19.8	96.2–96.2	45.4–45.4	48.4–48.4
	VL50	Absolute	0.0–0.0	1.0–1.0	0.5–0.5	0.73–0.73	0.4–0.4	8.1–8.1	12.6–12.6	–
		Relative	0.0–0.0	4.9–4.9	5.6–5.6	112.7–112.7	18.6–18.6	114.1–114.1	46.5–46.5	54.0–54.0
	VL65	Absolute	0.0–0.0	1.0–1.0	0.5–0.5	0.73–0.73	0.4–0.4	8.9–8.9	12.9–12.9	–
		Relative	0.0–0.0	7.8–7.8	5.0–5.0	110.8–110.8	16.6–16.6	127.6–127.6	47.8–47.8	57.0–57.0
CLUSTER 2. Early acceleration	OS	Absolute	0.7–1.8	2.0–2.9	4.7–5.4	0.16–0.18	1.2–1.4	5.4–6.4	15.4–16.7	–
		Relative	1.4–3.78	7.7–11.3	49.9–57.3	25.0–27.7	55.2–62.0	76.1–89.9	56.8–61.8	30.8–38.1
	UNRESISTED	Absolute	0.5–2.8	2.0–4.0	4.2–6.0	0.15–0.18	1.1–1.4	5.5–6.8	14.4–16.7	–
		Relative	1.12–5.84	7.3–14.5	45.1–63.3	22.4–27.5	47.7–61.2	78.1–97.4	53.5–61.9	31.6–41.9
	VL10	Absolute	0.5–3.4	2.0–4.5	4.0–5.9	0.15–0.19	1.0–1.4	5.2–6.6	13.7–16.4	–
		Relative	1.6–10.49	9.5–21.5	42.3–62.4	23.6–29.8	46.2–60.9	74.9–95.2	51.1–61.3	30.9–42.8
	VL25	Absolute	0.4–5.0	2.0–6.1	3.8–5.8	0.15–0.2	1.0–1.3	5.2–7.3	13.6–17.1	–
		Relative	1.47–16.71	9.1–27.5	39.8–61.0	23.1–31.2	44.0–59.5	73.9–102.7	50.1–63.0	30.2–46.4
	VL50	Absolute	0.4–20.5	2.0–20.1	3.0–5.1	0.16–0.24	0.8–1.3	5.1–7.9	13.2–17.8	–
		Relative	1.99–98.21	9.8–98.5	31.2–53.9	24.5–36.7	37.6–58.3	71.3–110.9	48.6–65.4	29.4–48.6
	VL65	Absolute	0.4–10.9	2.0–13.4	2.4–4.3	0.18–0.27	0.7–1.1	6.3–8.6	13.4–16.2	–
		Relative	3.39–100	15.7–100.0	25.6–45.2	27.6–41.9	33.4–51.9	89.2–121.2	49.4–59.7	38.1–51.5
CLUSTER 3. Mid-acceleration	OS	Absolute	3.2–10.0	3.8–7.9	6.1–8.1	0.11–0.15	1.5–1.8	3.1–4.8	18.3–22.7	–
		Relative	6.71–21.24	14.7–30.8	64.0–85.8	17.1–22.6	68.3–83.2	43.6–68.3	67.4–83.4	13.6–25.2
	UNRESISTED	Absolute	4.1–14.7	5.0–11.2	6.2–8.5	0.11–0.14	1.5–1.9	2.6–4.7	17.4–22.4	–
		Relative	8.76–31.17	18.2–41.2	66.2–89.8	16.6–22.1	64.9–83.9	37.5–67.0	64.8–83.2	12.0–25.3
	VL10	Absolute	4.7–20.2	5.5–14.9	6.1–8.2	0.11–0.15	1.4–1.9	2.5–4.7	17.0–22.2	–
		Relative	14.7–62.66	26.3–70.4	64.9–86.9	17.1–23.1	64.5–83.2	36.4–67.5	63.4–82.9	11.6–26.1
	VL25	Absolute	6.2–28.6	7.0–21.4	5.8–7.1	0.12–0.15	1.4–1.9	2.7–5.1	17.1–21.7	–
		Relative	20.71–95.49	31.7–96.5	60.9–75.4	18.8–23.6	62.8–84.9	38.1–71.9	63.3–80.3	12.8 to 27.1
CLUSTER 4. Late acceleartion	OS	Absolute	11.8–47.1	8.9–25.7	8.4–10.3	0.09–0.11	1.9–2.3	−1.1–2.6	23.0–27.9	–
		Relative	25.13–100	34.6–100.0	88.3–109.0	13.7–16.7	85.9–104.6	−15.7–37.0	84.6–103.0	−4.1–10.9
	UNRESISTED	Absolute	15.2–47.1	11.5–27.3	8.3–9.4	0.1–0.11	1.9–2.2	−0.2–2.4	22.3–26.7	–
		Relative	32.16–100	42.1–100.0	87.8–99.6	14.8–17.2	83.4–99.4	−3.8–34.5	83.0–99.3	−0.9–10.4
	VL10	Absolute	20.6–32.4	15.1–21.1	7.9–8.5	0.11–0.12	1.9–2.2	−0.6–2.4	22.4–25.7	–
		Relative	63.71–100	71.4–100.0	83.2–89.6	16.5–18.1	83.2–99.3	−9.0–34.5	83.3–95.6	−2.4–10.4
	VL25	Absolute	29.8–30.3	21.7–21.9	6.7–6.7	0.13–0.14	2.0–2.0	1.7–1.9	23.2–23.4	–
		Relative	98.33–100	98.8–100.0	70.3–71.0	19.8–20.3	88.0–88.9	24.3–27.0	84.4–85.1	7.4–8.2

Absolute units for the described values: distance (m); Step (*n*); Speed (m/s); contact time (s); step length (m); horizontal force (*N*/Kg); vertical force (*N*/Kg).

## 4 Discussion

This research explores the impact of varying overload conditions on the mechanical determinants of athletic performance during sprint acceleration. The findings suggest that these mechanical determinants may align with the distinct phases of sprinting documented in existing literature. Overload conditions, whether involving heavy or light loads, seem to recreate specific mechanical contexts. Heavy loads tend to produce more targeted stimuli, primarily affecting specific sprint phases due to rapid speed stabilization under exclusively high-force low-speed scenarios, whereas light loads seem to provide broader stimuli, influencing performance across a wider range of sprint phases.

Recent findings suggest that at a specific speed, variations in load minimally affect spatiotemporal variables and GRFs, indicating that running speed may be more influential than load (29). While speed is widely recognized as a key determinant of training intensity, it may not fully encompass/capture sprint mechanics under ecological conditions. This limitation is particularly evident when training stimulus is solely focused on the plateau phase of sprint runs.

Regardless of the load used, sprints typically exhibit a progressive decline in acceleration capability until maximum velocity is reached (14). Consequently, current methodologies based on velocity loss are most applicable to phases where velocity has stabilized. However, our findings suggest that the plateau phase (90%–100% of maximum velocity) accounts for only 20%–30% of the steps required to reach peak velocity depending on loading condition (see Figure 2). This observation implies that relying solely on the plateau phase for stimuli characterization might be particularly compromised when speed stabilization is delayed. To address current methodological limitations (12, 34, 35), this study employs cluster analysis to identify scenarios that best replicate the mechanical characteristics of free sprinting across various overload conditions. The categories derived from selected mechanical determinants appear to align closely with the sprint phases identified in the literature, such as block start, early acceleration, late acceleration, and top speed (31, 36).

Analysis of these clusters reveals consistent patterns across analyzed sprint runs, regardless of overload condition. Typically, the distribution of forces transitions from a balanced force ratio -around 50% at initial contact- to a gradual decline, primarily due to the rapid decrease in horizontal force application, while vertical forces steadily increase. This shift is often attributed to changes in body segment orientation that occur with increasing displacement speed (36, 37). Notably, although higher speeds result in reduced contact times, this is accompanied by a continuous increase in step length and stabilization or decline in step rate, indicating a dynamic adaptation of force application scenarios throughout the sprint (see Supplementary File S1).

Cluster 1, corresponding to initial contact data for each athlete, was characterized by longer contact times, greater horizontal force application, and higher force ratios compared to the subsequent clusters. Additionally, shorter step lengths and reduced vertical force applications were observed, regardless of the applied overload (Tables 2, 3). These step mechanics align with the findings of previous studies, which identified low to null displacement velocities and specific body arrangements as primary factors influencing kinetic and kinematic conditions (38–40). The consistent clustering of all initial contacts indicates a high level of specificity during this phase, suggesting that stimulating scenarios is not conditioned by the type of overload used, as the mechanical characteristics of these conditions are consistently reproduced across all analyzed scenarios (Figure 1).

**Figure 3:**
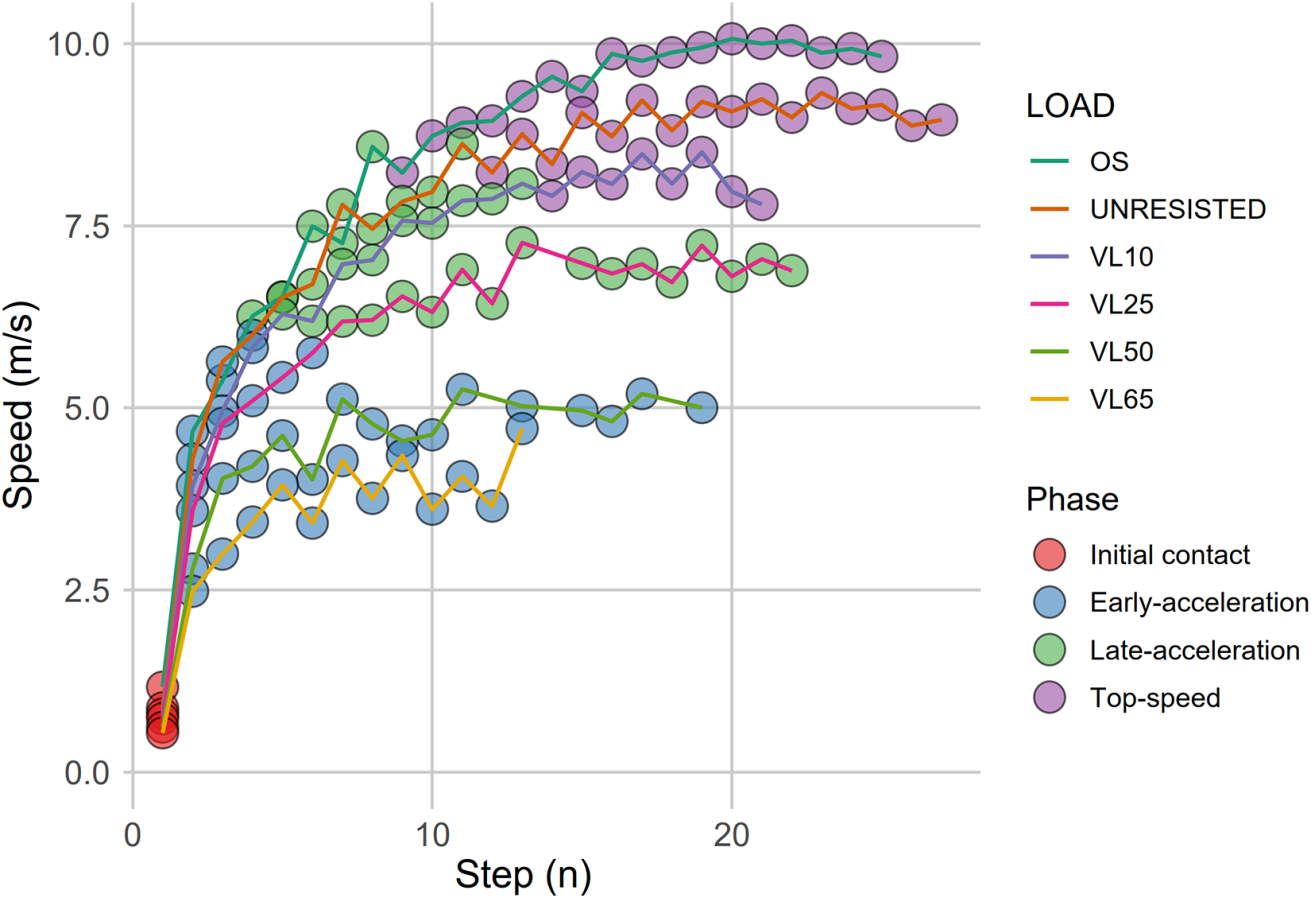
Step-specific stimuli under varying overloading conditions.

Horizontal force remains consistent with the preceding steps (*p* > 0.05) but differs from the following ones in Cluster 2. While horizontal force stays stable during the early steps of acceleration, RF decreases due to an increase in mean vertical force compared to initial contact steps (Cluster 1). This shift marks the beginning of a trend of progressive decline that persists throughout the entire acceleration curve. Separately, our analysis reveals that the number of steps recreating the mechanical characteristics of the initial acceleration phase (Cluster 2) is influenced by the type of overload applied. For lighter loads, such as OS, unresisted, or VL25, steps 2–4 fall into cluster 2. However, with heavier loads, particularly those classified as VL50 and VL65, this cluster extends to steps 7–8. This observation highlights a key distinction in the diverse training methodologies aimed at enhancing early-acceleration (27, 41). In this sense, some approaches recommend using light loads to avoid mechanical disturbances and maintain the natural sprint mechanics observed under free sprint conditions (11, 12, 22). These approaches, involving loads less than 20% of body weight or with velocity losses under 10%, are usually applied over short distances of 20 and 30 meters per set (42–45). However, while light loads are effective in preserving natural sprint mechanics, they may lack the specificity needed/required for the sustained replication of initial contact conditions. This reduced specificity, resulting from the rapid increase in velocity that light loads facilitate, could limit the effectiveness of these approaches compared to heavier loads, which allow for a more prolonged replication of the mechanical characteristics necessary for early acceleration (see Figures 2, 3).

Cluster 3 corresponds to steps in the middle acceleration phase, also referred as the transition phase, characterized by a marked decrease in horizontal force compared to the preceding clusters, aligning with the concept of a decreasing ratio of forces throughout the acceleration phase, as described by Morin et al. (7). While this cluster includes steps from all overload conditions, it primarily comprises contacts associated with the late acceleration phase of lighter overloads (OS, unresisted, VL10, and VL25) and the plateau phase of VL10 and VL25. Although cluster spread varies depending on the overload type, similar onset points can be identified, typically occurring around the fourth or fifth step. This observation aligns with previous findings by Nagahara et al. (31, 36), who reported the transition phase beginning around 4–5 steps under free-sprint conditions in elite sprinters. As the overload increases, the transition to the next cluster is delayed, with the upper limits extending from steps 9–10 under OS and unresisted conditions to potentially encompassing the entire plateau phase for VL25 loads. This delay indicates that heavier overloads prolong the scenario typically observed during the free-sprint acceleration phase, potentially enhancing the training stimulus for this crucial phase of sprinting (46). By understanding these dynamics, coaches can strategically select and adjust overloads, considering both the specific conditions and the steps required to optimally target and prolong the transition phase, thereby maximizing training effectiveness.

Cluster 4 corresponds to steps associated with mechanical factors typically observed during free-sprint maximum velocity phases. Our analysis reveals that only light overloads (OS, unresisted, and late VL10 steps) successfully replicate conditions featuring reduced contact times (0.09–0.14s) and predominantly vertical force orientation at very high speeds (RF ranging from 10% to 0%), resulting in significantly longer step distances (Table 3). These scenarios, however, are only reproduced from the 11- to 29-meter mark onwards, depending on the overload applied. This variation can substantially affect the effectiveness of the training stimulus over a given distance and exposes the limitations of training prescriptions or analyses that focus exclusively on the sprint plateau phases, which account for only a small portion of overall sprint performance. These findings underscore the importance of identifying and prioritizing specific training phases, as distinct overloads have unique impacts on sprint mechanics at different points in the sprint distance.

In this sense, a central challenge in performance training is determining which specific attributes or performance phases should be prioritized for improvement. For sprint coaches, this often begins with an analysis of the velocity-time curve to pinpoint the acceleration phase most in need of targeted development. Under the principle of specificity, it is hypothesized that stimuli closely aligned with the athlete's target movement contexts may yield more efficient and relevant adaptations. Within this framework, strategies that incorporate sustained velocity loss have been proposed as a means to enhance exposure to these specific contexts, thereby fostering adaptations linked to selected movement velocities.

Our findings support this hypothesis by demonstrating that controlled loads effectively recreate contexts with a high degree of similarity to targeted sections of the velocity-time curve. However, they also reveal previously unconsidered aspects, such as the point at which a prescribed load begins to meaningfully stimulate the intended section. When examining two commonly discussed examples in the literature -early and late acceleration phases- our analysis confirms that once movement velocity stabilizes (i.e., the plateau phase), stimuli are consistently reproduced according to the intended training context. For instance, loads at 50% and 65% velocity loss (VL50 and VL65) effectively target early acceleration, while lighter loads [e.g., overspeed [OS], free sprinting [FS], and 10% velocity loss [VL10]] align well with conditions for later stages of acceleration.

However, by examining these stimuli through the lens presented in this study, distinct variations emerge in the behavior of movement prior to reaching stabilized speed. Although any overload will inevitably include the initial step due to its unique characteristics, heavier loads appear to replicate the demands that characterize early acceleration in free conditions from the second step onward, even without reaching plateau values (typically achieved around steps 8–9 under these conditions). This observation may support the high effectiveness of heavy loads for stimulating high-force, low-velocity contexts, as highlighted in the literature.

**Figure 4:**
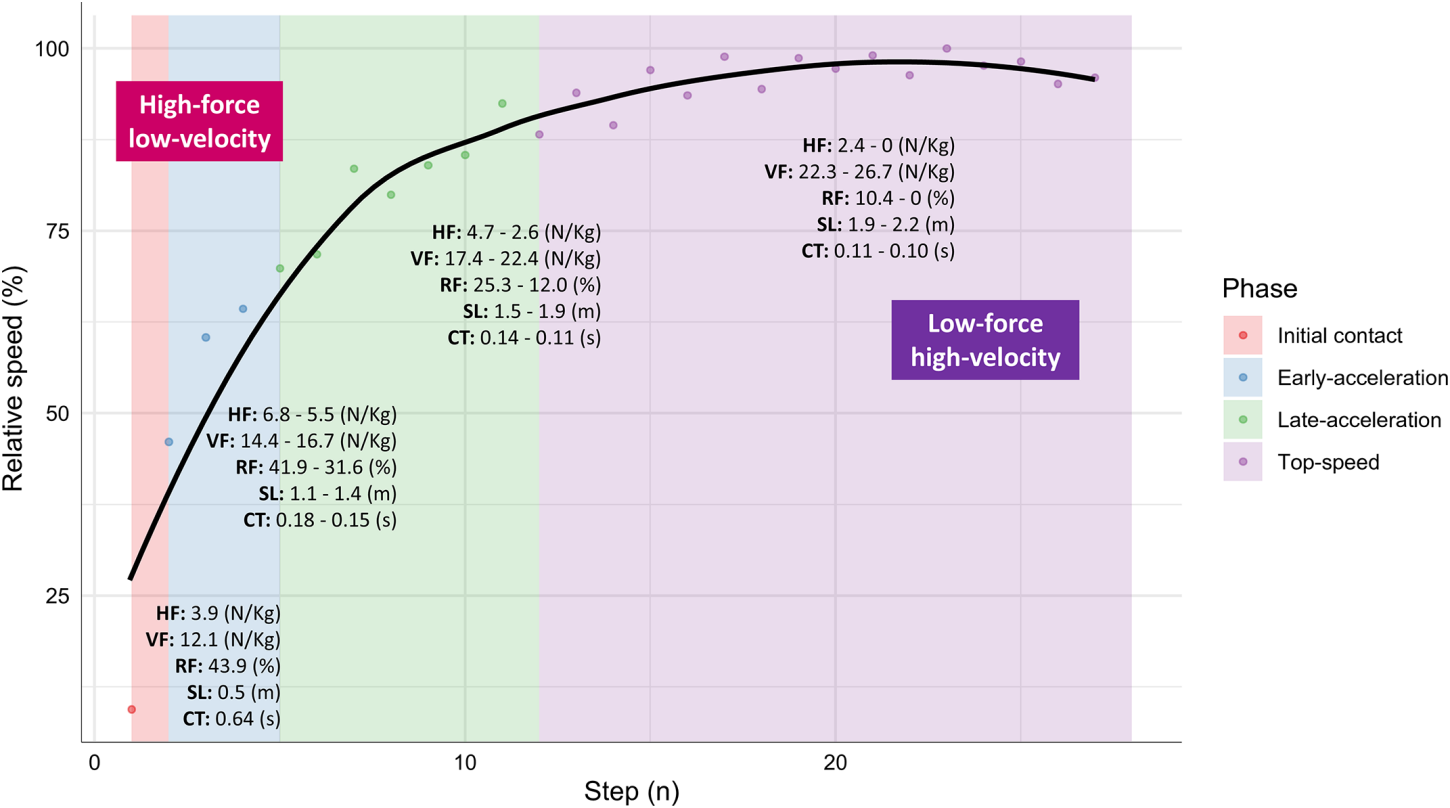
Cluster-specific kinetic and kinematic profiles during free sprinting.

On the other hand, lighter loads also reach a stabilized context for training but do so much later, reducing the proportion of steps or distance covered per repetition that effectively targets early acceleration. Based on our findings, lighter loads (such as OS, FS, and VL10) seem to replicate a broader range of stimulation contexts and are particularly valuable for training low-force, high-velocity scenarios. Under these conditions, the stabilization of recreated contexts occurs around steps 9–15 (see Table 1), which closely align with the moments at which plateau phase is reached under analyzed conditions (steps 10–12) (see Figures 2–4).

From a practical perspective, the results not only offer valuable insights into the effectiveness of various overload conditions in replicating the mechanical characteristics critical to sprint performance but also help identify the most effective training stimuli. Furthermore, the discussion enables a more detailed characterization of the training stimulus beyond the plateau phases, thereby offering important insights into how training volume per set is influenced by the chosen overload condition. Thus, monitoring the number of steps associated with each scenario would provide coaches with an alternative tool for the objective quantification of the periodization models applied throughout the season, based on criteria of specificity. For instance, when examining specificity in recreating targeted mechanical contexts within a standard early acceleration session covering 20 meters per set, heavy loads (e.g., VL50 and VL65) replicate the desired phase for a significant portion of the distance (approximately 19 out of 20 steps or nearly the full 20 meters). In contrast, lighter loads (e.g., VL10) recreate the targeted scenario for only 2–3 steps (roughly 3 meters when analyzing the average distance covered under this overload conditions for early acceleration) within the 15 steps required to complete the 20-meter distance (see Table 3).

The findings present coaches and practitioners with a refined approach to monitoring and adjusting training across distinct phases of sprint performance. Through “phase-specific sprinting exposure,” coaches gain a deeper understanding of how targeted the stimuli are throughout all acceleration phases, from early to late stages. This perspective supports a more precise approach to load monitoring, drawing parallels to methods in velocity-based training by emphasizing the analysis of velocity range distribution across each repetition. Such an approach allows for a more systematic assessment of sprint-specific stimulation, potentially enhancing the effectiveness and specificity of resistance-based sprint training.

Future research should investigate the long-term training effects of manipulating overload conditions to target specific sprint phases to verify if the periodic recreation of acute training stimuli leads to the desired long-term adaptation.

## Data Availability

The raw data supporting the conclusions of this article will be made available by the authors, without undue reservation.
