# Supplementary figures and images for: Understanding sprint phase-specific training stimuli: a cluster analysis approach to overload conditions
Source: Front Sports Act Living. 2024 Dec 10;6:1510379. doi: 10.3389/fspor.2024.1510379 (PMC11669057; doi:10.3389/fspor.2024.1510379)

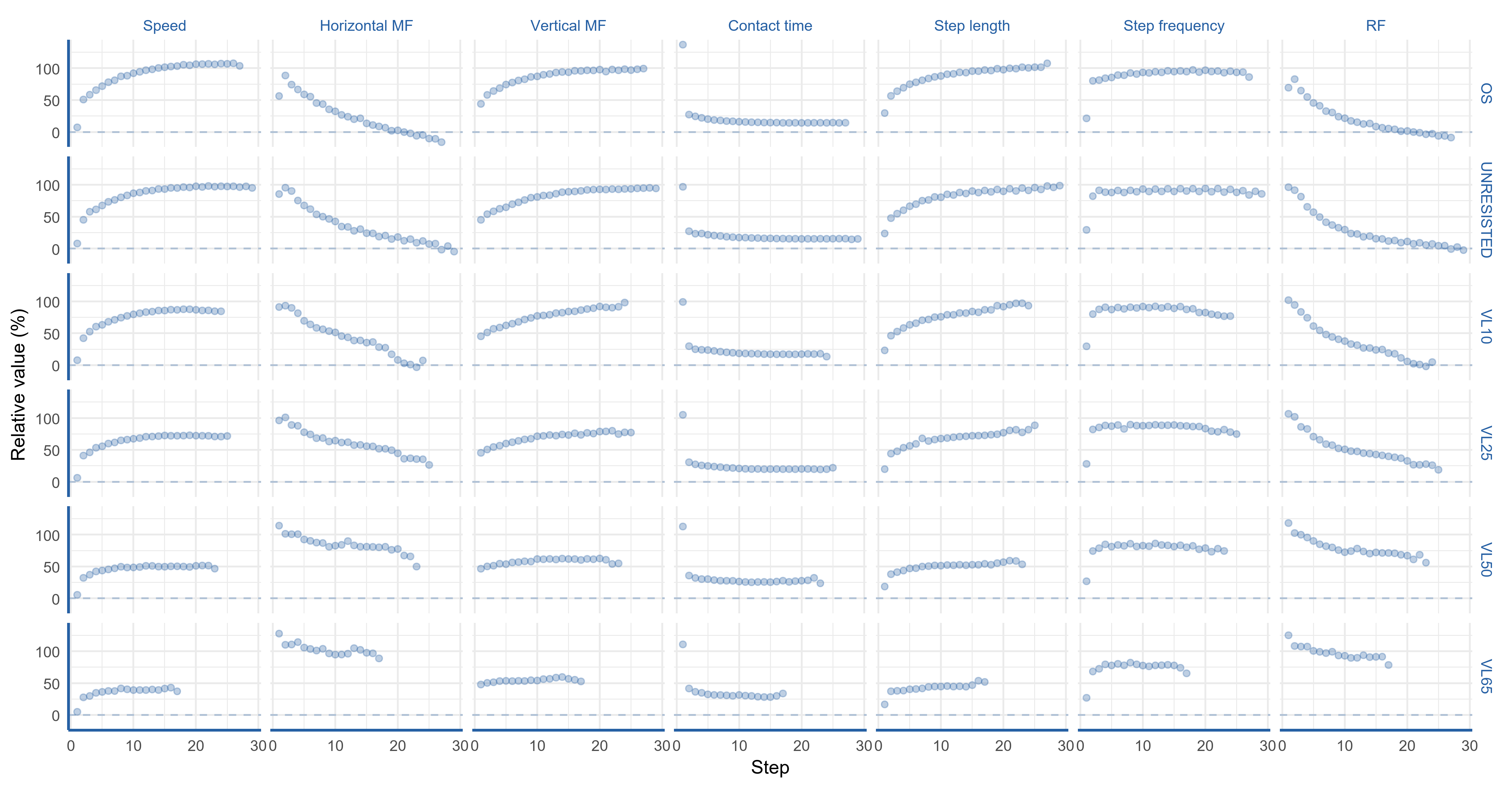

Supplement: Supplementary file 1 [file Image1.tif]
