# Supplementary material for: Understanding sprint phase-specific training stimuli: a cluster analysis approach to overload conditions
Source: Front Sports Act Living. 2024 Dec 10;6:1510379. doi: 10.3389/fspor.2024.1510379 (PMC11669057; doi:10.3389/fspor.2024.1510379)
Supplement: Supplementary file 2 [file Table1.docx]

|  |  | **Step frequency [Hz]** | **Power [W/kg]** | **Flight time [s]** | **Brak MF [N/kg]** | **Vert Imp [Ns/kg]** | **AP net Imp [Ns/kg]** | **Prop Imp [Ns/kg]** | **Brak Imp [Ns/kg]** |
| --- | --- | --- | --- | --- | --- | --- | --- | --- | --- |
| *Initial contact* | OS | 1.0 to 1.0 | 2.7 to 2.7 | 0.1 to 0.1 | -0.6 to -0.6 | 10.7 to 10.7 | 3.4 to 3.4 | 3.6 to 3.6 | -0.2 to -0.2 |
|  | UNRESISTED | 1.4 to 1.4 | 4.5 to 4.5 | 0.1 to 0.1 | -0.0 to -0.0 | 7.7 to 7.7 | 3.8 to 3.8 | 3.8 to 3.8 | -0.0 to -0.0 |
|  | VL10 | 1.4 to 1.4 | 4.7 to 4.7 | 0.1 to 0.1 | -0.0 to -0.0 | 7.8 to 7.8 | 4.1 to 4.1 | 4.1 to 4.1 | -0.0 to -0.0 |
|  | VL25 | 1.4 to 1.4 | 4.0 to 4.0 | 0.1 to 0.1 | -0.0 to -0.0 | 8.4 to 8.4 | 4.6 to 4.6 | 4.6 to 4.6 | -0.0 to -0.0 |
|  | VL50 | 1.3 to 1.3 | 4.2 to 4.2 | 0.0 to 0.0 | -0.0 to -0.0 | 9.2 to 9.2 | 5.9 to 5.9 | 5.9 to 5.9 | -0.0 to -0.0 |
|  | VL65 | 1.3 to 1.3 | 4.2 to 4.2 | 0.0 to 0.0 | -0.0 to -0.0 | 9.5 to 9.5 | 6.5 to 6.5 | 6.5 to 6.5 | -0.0 to -0.0 |
| *Early acceleration* | OS | 3.8 to 4.0 | 27.9 to 31.7 | 0.1 to 0.1 | -1.6 to -0.3 | 2.7 to 2.8 | 0.9 to 1.2 | 0.9 to 1.2 | -0.0 to -0.0 |
|  | UNRESISTED | 3.9 to 4.6 | 27.1 to 35.8 | 0.1 to 0.1 | -3.3 to -1.1 | 2.3 to 2.7 | 0.8 to 1.2 | 0.9 to 1.2 | -0.1 to -0.0 |
|  | VL10 | 3.9 to 4.5 | 25.8 to 33.8 | 0.1 to 0.1 | -3.9 to -1.4 | 2.4 to 2.7 | 0.8 to 1.3 | 0.9 to 1.3 | -0.1 to -0.0 |
|  | VL25 | 3.8 to 4.5 | 23.1 to 34.7 | 0.0 to 0.1 | -4.1 to -0.6 | 2.4 to 2.8 | 0.8 to 1.4 | 0.9 to 1.4 | -0.1 to -0.0 |
|  | VL50 | 3.4 to 4.4 | 19.5 to 34.2 | 0.0 to 0.1 | -3.6 to -0.2 | 2.6 to 3.2 | 0.9 to 1.7 | 0.9 to 1.7 | -0.1 to -0.0 |
|  | VL65 | 3.2 to 4.3 | 17.7 to 33.2 | 0.0 to 0.1 | -2.2 to 0.0 | 2.7 to 3.7 | 1.3 to 2.1 | 1.3 to 2.1 | -0.0 to 0.0 |
| *Mid acceleration* | OS | 4.0 to 4.5 | 24.3 to 31.2 | 0.1 to 0.1 | -3.2 to -1.4 | 2.4 to 2.8 | 0.4 to 0.7 | 0.4 to 0.7 | -0.1 to -0.0 |
|  | UNRESISTED | 4.2 to 4.7 | 21.4 to 31.3 | 0.1 to 0.1 | -4.1 to -2.3 | 2.3 to 2.6 | 0.3 to 0.7 | 0.4 to 0.7 | -0.1 to -0.0 |
|  | VL10 | 4.2 to 4.6 | 20.2 to 31.1 | 0.1 to 0.1 | -4.7 to -2.3 | 2.3 to 2.7 | 0.3 to 0.7 | 0.4 to 0.7 | -0.1 to -0.0 |
|  | VL25 | 3.7 to 4.5 | 18.4 to 33.2 | 0.1 to 0.1 | -4.6 to -2.0 | 2.4 to 2.9 | 0.4 to 0.7 | 0.5 to 0.8 | -0.1 to -0.0 |
| *Late acceleration* | OS | 4.2 to 4.8 | -10.7 to 22.4 | 0.1 to 0.1 | -7.3 to -3.0 | 2.3 to 2.7 | -0.1 to 0.3 | 0.2 to 0.4 | -0.3 to -0.1 |
|  | UNRESISTED | 4.0 to 4.7 | -1.9 to 20.8 | 0.1 to 0.1 | -6.0 to -3.0 | 2.3 to 2.7 | -0.0 to 0.3 | 0.3 to 0.4 | -0.3 to -0.1 |
|  | VL10 | 3.7 to 4.4 | -4.7 to 20.1 | 0.1 to 0.2 | -5.8 to -3.0 | 2.5 to 2.9 | -0.1 to 0.3 | 0.2 to 0.4 | -0.3 to -0.1 |
|  | VL25 | 3.4 to 3.4 | 11.6 to 13.0 | 0.2 to 0.2 | -2.9 to -2.7 | 3.1 to 3.2 | 0.2 to 0.3 | 0.4 to 0.4 | -0.1 to -0.1 |
